# Effect of transcription inhibition and generation of suppressive viral non-coding RNAs

**DOI:** 10.1186/s12977-019-0475-0

**Published:** 2019-04-29

**Authors:** Daniel O. Pinto, Tristan A. Scott, Catherine DeMarino, Michelle L. Pleet, Thy T. Vo, Mohammed Saifuddin, Dmytro Kovalskyy, James Erickson, Maria Cowen, Robert A. Barclay, Chen Zeng, Marc S. Weinberg, Fatah Kashanchi

**Affiliations:** 1 Laboratory of Molecular Virology, School of Systems Biology, George Mason University, Manassas, VA USA; 2 Center for Gene Therapy, Beckman Research Institute of City of Hope, Duarte, CA USA; 3 Protein Engineering Department, Institute of Molecular Biology and Genetics, UAS, Kiev, Ukraine; 4 Department of Physics, The George Washington University, Washington, DC USA; 5 Department of Molecular and Experimental Medicine, The Scripps Research Institute, La Jolla, CA USA; 6 Wits/SA MRC Antiviral Gene Therapy Research Unit, Department of Molecular Medicine and Haematology, University of the Witwatersrand, Johannesburg, South Africa; 7 Laboratory of Molecular Virology, George Mason University, Discovery Hall Room 182, 10900 University Blvd., Manassas, VA 20110 USA

**Keywords:** HIV-1, Transcription, Latency, Gene silencing, cART, CRISPR

## Abstract

**Background:**

HIV-1 patients receiving combination antiretroviral therapy (cART) survive infection but require life-long adherence at high expense. In chronic cART-treated patients with undetectable viral titers, cell-associated viral RNA is still detectable, pointing to low-level viral transcriptional leakiness. To date, there are no FDA-approved drugs against HIV-1 transcription. We have previously shown that F07#13, a third generation Tat peptide mimetic with competitive activity against Cdk9/T1-Tat binding sites, inhibits HIV-1 transcription in vitro and in vivo.

**Results:**

Here, we demonstrate that increasing concentrations of F07#13 (0.01, 0.1, 1 µM) cause a decrease in Tat levels in a dose-dependent manner by inhibiting the Cdk9/T1-Tat complex formation and subsequent ubiquitin-mediated Tat sequestration and degradation. Our data indicate that complexes I and IV contain distinct patterns of ubiquitinated Tat and that transcriptional inhibition induced by F07#13 causes an overall reduction in Tat levels. This reduction may be triggered by F07#13 but ultimately is mediated by TAR-*gag* viral RNAs that bind suppressive transcription factors (similar to 7SK, NRON, HOTAIR, and Xist lncRNAs) to enhance transcriptional gene silencing and latency. These RNAs complex with PRC2, Sin3A, and Cul4B, resulting in epigenetic modifications. Finally, we observed an F07#13-mediated decrease of viral burden by targeting the R region of the long terminal repeat (HIV-1 promoter region, LTR), promoting both paused polymerases and increased efficiency of CRISPR/Cas9 editing in infected cells. This implies that gene editing may be best performed under a repressed transcriptional state.

**Conclusions:**

Collectively, our results indicate that F07#13, which can terminate RNA Polymerase II at distinct sites, can generate scaffold RNAs, which may assemble into specific sets of “RNA Machines” that contribute to gene regulation. It remains to be seen whether these effects can also be seen in various clades that have varying promoter strength, mutant LTRs, and in patient samples.

**Electronic supplementary material:**

The online version of this article (10.1186/s12977-019-0475-0) contains supplementary material, which is available to authorized users.

## Background

Retroviruses appear to be simple yet perform complex functions. They integrate into host chromosomal DNA and utilize the host’s replication machinery. The retrovirus human immunodeficiency virus-1 (HIV-1) has been heavily studied in recent years, yet no permanent cure has been discovered. Epidemiological data estimates about 36.7 million people worldwide are infected with HIV-1 and about 2 million new infections occur yearly [27]. There are multiple complications associated with chronic infection, such as HIV-1 associated neurocognitive disorders (HAND), which encompasses neurocognitive impairment in about 50% of patients despite the use of combination antiretroviral therapy (cART) [23, 26, 30, 34]. This chronic state, especially under cART, promotes a viral state of latency that may be represented by low level manifestations of viral products [7, 18]. Therefore, it is critical to not only understand the basic mechanisms of pathogenesis but also discover new treatments to combat the virus.

Resting T-cells or myeloid cells that are quiescent have been shown to allow a state of latency [41, 56, 71] with short bursts of small transcripts during a low or basal transcriptional state [1]. However, to date, there are no FDA-approved drugs against latency in treatment of HIV-1/AIDS patients. For full-length HIV-1 transcription, a combination of stimuli, such as T-cell activation and translation of the viral protein Tat, must occur. Tat is synthetized from a doubly spliced message which is initially able to be transcribed after T-cell receptor (TCR) activation via co-stimulation of CD3 and CD28 [38]. Following an initial round of transcription, Tat and NF-κB driven transcription generate mRNA production through both initiation and elongation; this is accomplished by Tat binding to TAR and recruitment of positive transcription elongation factor b (P-TEFb) [14, 41, 48]. This interaction results in the activation of P-TEFb kinase complex and phosphorylation of RNA Polymerase II (Pol II). The hyperphosphorylated Pol II is then able to read through nucleosomes containing chromatin complexes and stop at the 3′ LTR. The activation signals through the TCR, as well as ERK1/2, aid in de novo Tat synthesis and elicit transcriptional elongation [38].

Approximately half of those individuals infected with HIV-1 receive cART, which is typically comprised of a cocktail of inhibitors which target viral processes including entry, reverse transcription, integration, and protease-mediated cleavage [20, 32, 35, 44, 77]. Nevertheless, these therapies are ineffective at eradicating HIV-1. This is due to several reasons including lack of strict patient adherence to complex drug regimens, the development of viral resistance over time, inefficient and inconsistent penetration into tissues including the central nervous system (CNS) which thereby contributes to the formation of latent viral reservoirs, and the lack of specific transcriptional inhibitors in the treatment regimen [60, 65, 77].

To date there are at least six mechanism of HIV-1 transcriptional latency, which include: binding and sequestration of NF-κB in the cytoplasm, epigenetic silencing of Nuc-1 region at the transcriptional start site, transcriptional interference with Pol II from upstream or antisense promoters, sequestration of P-TEFb in the nucleus through 7SK RNA, BRD4/Tat competition for the HIV-1 promoter, and transcriptional silencing through non-coding viral RNA [1, 5, 24, 48, 63, 72]. Specifically, P-TEFb binding to Tat can initiate transcription [6] as well as recruit the super elongation complex (SEC), which contains ELLI, AFFI, ENL, AF9, and PAFc [14, 42, 76]. The P-TEFb/Tat complex enhances transcription by improving the processivity of Pol II [71] but can be disrupted by BRD4 through competitive inhibition of the Tat-binding site [8].

The HIV-1 LTR recruits proteins that contribute to nucleosome assembly and epigenetic silencing, although most Pol II molecules that are paused produce short transcripts that could serve as substrates for Dicer processing and micro-RNA (miRNA) generation [4, 39, 45, 59]. Similarly, long non-coding RNAs (lncRNAs) can also initiate latency by various mechanisms including epigenetic modifications, chromatin remodeling, and transcriptional silencing, among others [51, 62]. For instance, T-cells express a lncRNA known as NRON, which binds and degrades Tat, contributing to HIV-1 latency [43]. Also, 7SK small nuclear RNA (snRNA) sequesters P-TEFb, thereby preventing transcription [40].

Our laboratory has previously shown that small Tat peptides as well as ATP analogs can either compete for Tat binding or bind to Cdk9, resulting in inhibition of transcription. The Tat peptide mimetic F07#13 and ATP analog CR8#13, as well as Flavopiridol, inhibit transcription both in vitro and in vivo [15, 66, 68, 69]. F07#13 and CR8#13 were effective in cell lines as well as primary cells with low toxicity and transcriptional inhibition of multiple HIV-1 clades [12, 69].

In this manuscript, we have extended our previous findings on F07#13 and show that this peptide mimetic is able to inhibit elongation by Pol II and allow an increase of a novel form of HIV-1 non-coding RNA (TAR-*gag*). A study describing this RNA has previously been published by our laboratory [1, 7]. Similar to cellular RNAs, such as NRON, 7SK, HOTAIR, and Xist, transcriptional gene silencing (TGS) and latency of HIV-1 is promoted by the binding of viral lncRNAs to novel cellular targets [1]. The new RNA/protein complexes may allow multiple functions including methylation of histone tails (i.e. through PRC2), chromatin compaction (i.e. through Sin3A), and degradation of Tat (i.e. through Cul4B). This mode of action from the viral (or cellular) non-coding RNAs (ncRNA) resemble other RNA-assembled structure, such as ribosomes, where RNA serves as a scaffolding molecule to create a specific set of “RNA machines” that can potentially regulate transcription and DNA biology. Implications of these findings will be further discussed in the “Results” and “Discussion” sections.

## Results

### Effect of F07#13 on Tat levels

**Fig. 1:**
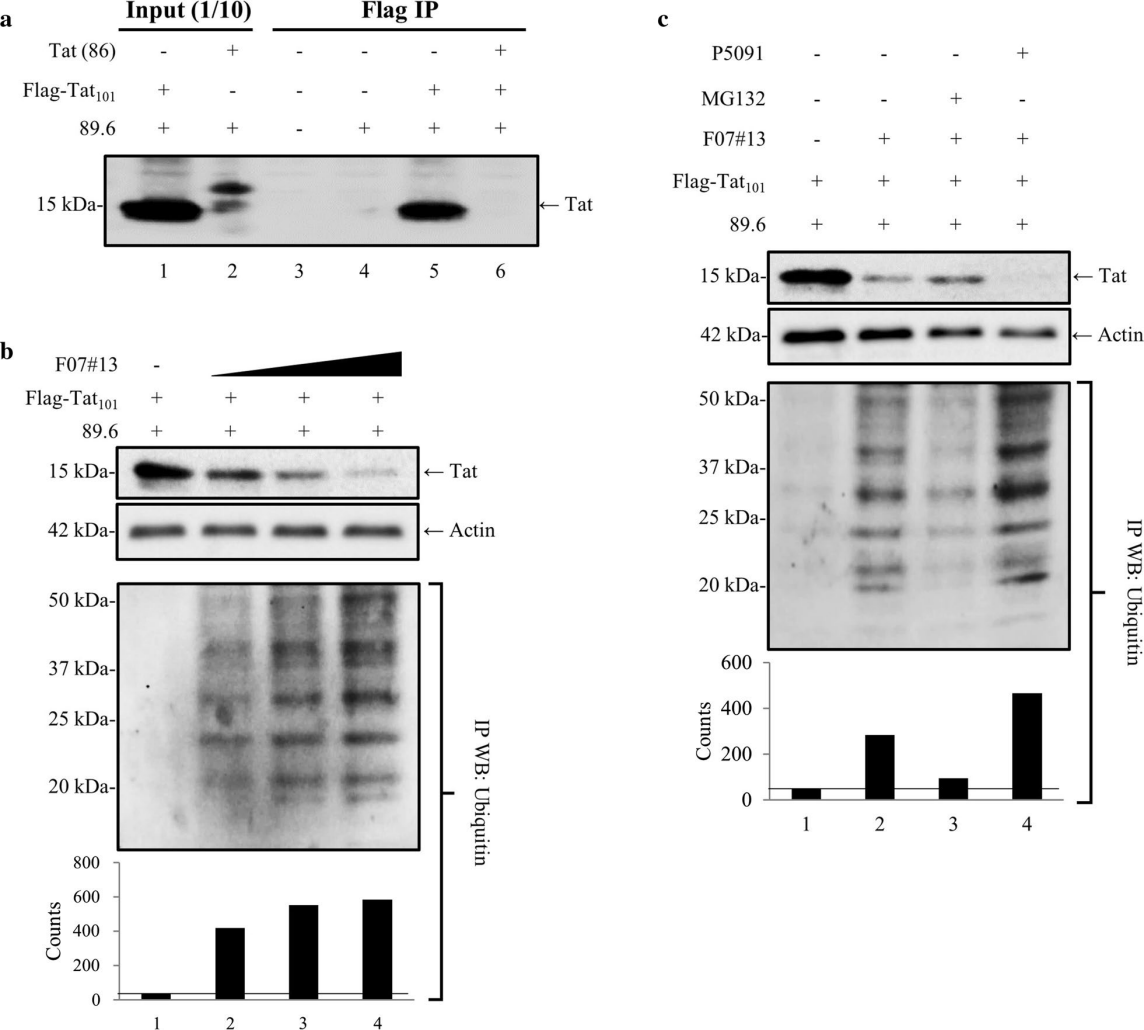
Effect of F07#13 on Tat degradation. **a** Following transfection into Jurkat cells, samples were collected and lysates were prepared for immunoprecipitation. Anti-Flag Ab was used for IP overnight, Protein A/G added the next day, washed, and samples were run on a gel and analyzed by Western blot for presence of Tat (α-Tat polyclonal Ab). Lanes 1 and 2 serve as control input transfected lysates (1/10) prior to IP. **b** Jurkat cells were transfected with 89.6 plasmid (20 μg) and CMV-Flag-Tat_101_ (20 μg) and 24 h later samples were treated with 0.01, 0.1, and 1 μM of F07#13 for an additional 48 h (a total 72 h). Cells were pelleted and washed, and lysates were run on a 4–20% Tris–glycine gel followed by Western blot with α-Flag antibody, followed by α-actin as a control. An IP with α-Flag antibody was run on a gel and probed with α-ubiquitin antibody. Densitometry was performed for each lane. **c** Cells were transfected with both 89.6 and Tat vector, followed by treatment with F07#13 (48 h; 1 μM) and two other inhibitors, MG132 (10 ng/mL) and a de-ubiquitin USP7 inhibitor (P5091; 3 μM), for 24 h and then separated on 4-20% Tris–glycine gel followed by Western blot with α-Flag antibody, α-ubiquitin antibody, and α-actin. Densitometry was performed to visualize changes in protein expression. The quantitation of 5 distinct bands in each lane was performed and summed to obtain total densitometry counts

HIV-1 Tat exists in multiple distinct complexes (large, medium, and small) in cells where the small complex contains Tat and Cdk9/T1 protein complex [69]. The small complex is believed to be important for HIV-1 Tat activated transcription and is detected in a number of cell lines and primary infected cells [9, 10, 40, 47]. Here, we first examined whether F07#13 had any effect on Tat levels in cells. Therefore, we transfected Jurkat cells with a dual-tropic wild-type viral construct (89.6) along with a Tat plasmid (CMV-Flag-Tat_101_). We have previously used this Tat construct in transfections and were able to detect Tat levels in cells using Flag antibody [3, 22]. Results of such an experiment are shown in Fig. 1a where Tat was specifically immunoprecipitated only when anti-Flag antibody was used. A construct of Tat, Tat (86), which did not contain Flag-Tag, was not precipitated under these conditions (compare lanes 3 and 4). We next explored the effect of F07#13 on Tat levels and found that Tat was present in detectable amounts; however, when F07#13 (0.01, 0.1, 1 µM) was added to cells 24 h post-transfection with Flag-Tat_101_ or 89.6 and incubated for 48 h with F07#13, the levels of Tat decreased with increasing concentration of F07#13 (Fig. 1b). We were surprised by these results as the Tat vector is driven by a CMV promoter and is not regulated by F07#13 or other inhibitors except NF-κB inhibitors or Flavopiridol [11, 52, 57] (data not shown). Along these lines, when using CMV-Tax as a control (transactivator from HTLV-1), we observed no changes in Tax levels in the presence of F07#13, indicating that F07#13 was specific to Tat and not the CMV promoter (Additional file 1: Fig. S1). We therefore reasoned that Tat may be modified (i.e. ubiquitinated) which could target Tat for degradation. Along these lines, Tat has previously been shown to be modified by us and others including acetylation, methylation, and ubiquitination [19, 54, 58, 70].

We next probed for the presence of ubiquitinated-Tat (Ub-Tat) using Flag immunoprecipitated (IP) followed by Western blot for presence of ubiquitin conjugates. Results in bottom panel of Fig. 1b show that increasing concentration of F07#13 resulted in detection of Ub-Tat, indicating that Tat may potentially be modified. Quantification of total ubiquitinated proteins are graphed underneath the blot. Also, a recent report has shown that Tat is stabilized by the de-ubiquitinase USP7, leading to enhanced viral production [2]. Therefore, we performed another similar experiment with the addition of a proteasome inhibitor (MG132) and a de-ubiquitin inhibitor (P5091). Cells were transfected with both 89.6 and the Flag-Tat_101_ vector and then treated with F07#13 (1 µM) for 48 h. Twenty-four hours post F07#13 treatment, cells were treated with MG132 (10 ng/mL) or P5091 (3 µM) and incubated for 24 h. Results in Fig. 1c show that Tat levels were decreased with F07#13; however, addition of P5091 greatly reduced Tat levels. As a follow-up, we performed a Western blot with α-ubiquitin antibody and observed an increased level of Ub-Tat when using P5091. Quantifications of total ubiquitinated proteins are graphed below the blot. Collectively, these data indicate that F07#13 treated cells, in which interactions between Tat and Cdk9/T1 complexes are inhibited [69], have enhanced Tat-ubiquitination and potential degradation.

### Effects of F07#13 on various Tat complexes

**Fig. 2:**
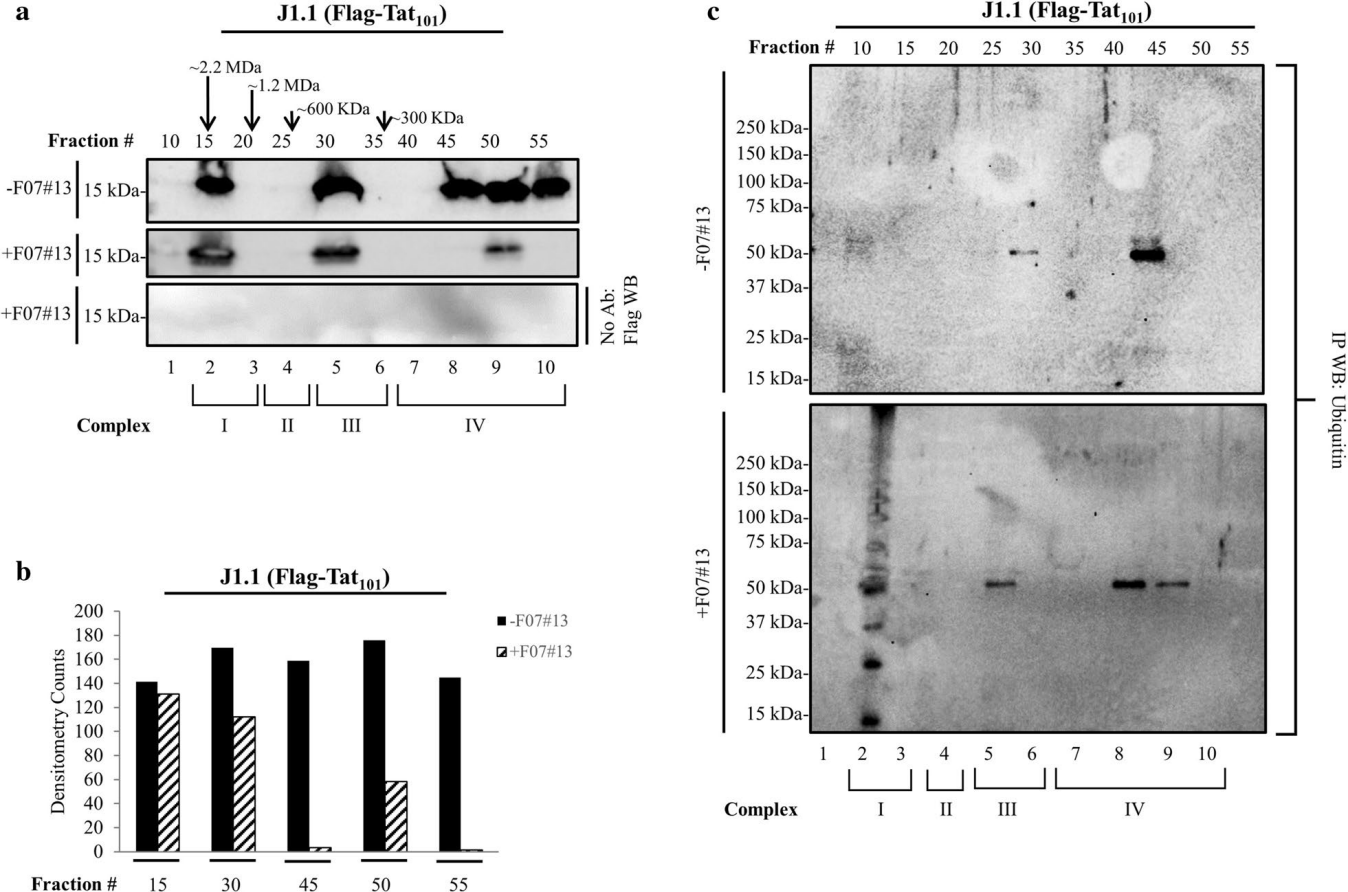
Presence of ubiquitin-Tat in the large complex. **a** HIV-1 infected J1.1 cells were electroporated with CMV-Flag-Tat_101_ (20 μg) and kept at 37 °C for 48 h. Cells were isolated, washed, and extracts were processed for FPLC chromatography (Superose 6) using high salt. A total of 3.5 mg was used for chromatography. Flow rate parameters for the FPLC were set at 0.3 mL/min and 0.5 mL fractions of the flow-through were collected at 4 °C for approximately 60 fractions per sample (1 mL injected). Tat associated complexes were nanotrapped with NT084 and assayed for Western blot using α-Flag antibody. **b** Densitometry counts from panel a were obtained, normalized to background, and plotted to represent the relative abundance of Tat protein in each fraction. **c** Chromatography fractions were IPed with α-Flag antibody overnight, followed by addition of Protein A/G, ran on a gel, and analyzed by Western blot with α-ubiquitin antibody. Two sets of extracts (± F07#13) were run on chromatography and used for nanotrapping and Western blots

We next examined the effect of F07#13 on three distinct Tat complexes using J1.1 cells. Here, we used electroporation to transfect Flag-Tat_101_ into J1.1 cells (containing wild type virus) and obtained whole cell extracts for fractionation using fast protein liquid chromatography (FPLC). We have previously used this method to separate Tat associated complexes (4 distinct complexes) using Flag-Tat antibody from infected cells under high salt conditions [1, 69]. The fractions were further concentrated using nanoparticles (NT084) and run on a 4–20% Tris–glycine gel for Western blot analysis. Results in Fig. 2a show that Tat separated into three distinct fractions ranging from small molecular weight (< 300 kDa; lanes 8–10) to medium size (300–600 kDa; lane 5) and large molecular weight (1.2–2.2 MDa; lane 2) complexes. These complexes were previously designated as Complexes I-IV from these infected cells [69]. However, complex II did not contain any Tat protein. Importantly, F07#13 treated cells exhibited a disappearance of most of the small molecular weight complexes (Complex IV). Control IP without antibody was used for these fractions (Protein A/G only), followed by Western blot with anti-Flag antibody. Total quantification of each fraction in the presence and absence of F07#13 is shown in Fig. 2b. We then treated the IP blots with α-ubiquitin antibody and observed a distinct pattern of ubiquitination in one of the lanes for Complex IV (Fig. 2c; lane 9), but a more prominent pattern of ubiquitination for Complex I (Fig. 2c; lane 2). This pattern of ubiquitination has previously been observed for Tat and a number of other viral transactivators [2, 13, 16, 33, 61, 64, 67, 73]. Collectively, these data indicate that Tat ubiquitination may be regulated by F07#13, resulting in lower levels of Tat in treated cells.

### Presence of novel HIV-1 ncRNA (TAR-*gag*) in large complexes co-sedimenting with Tat

**Fig. 3:**
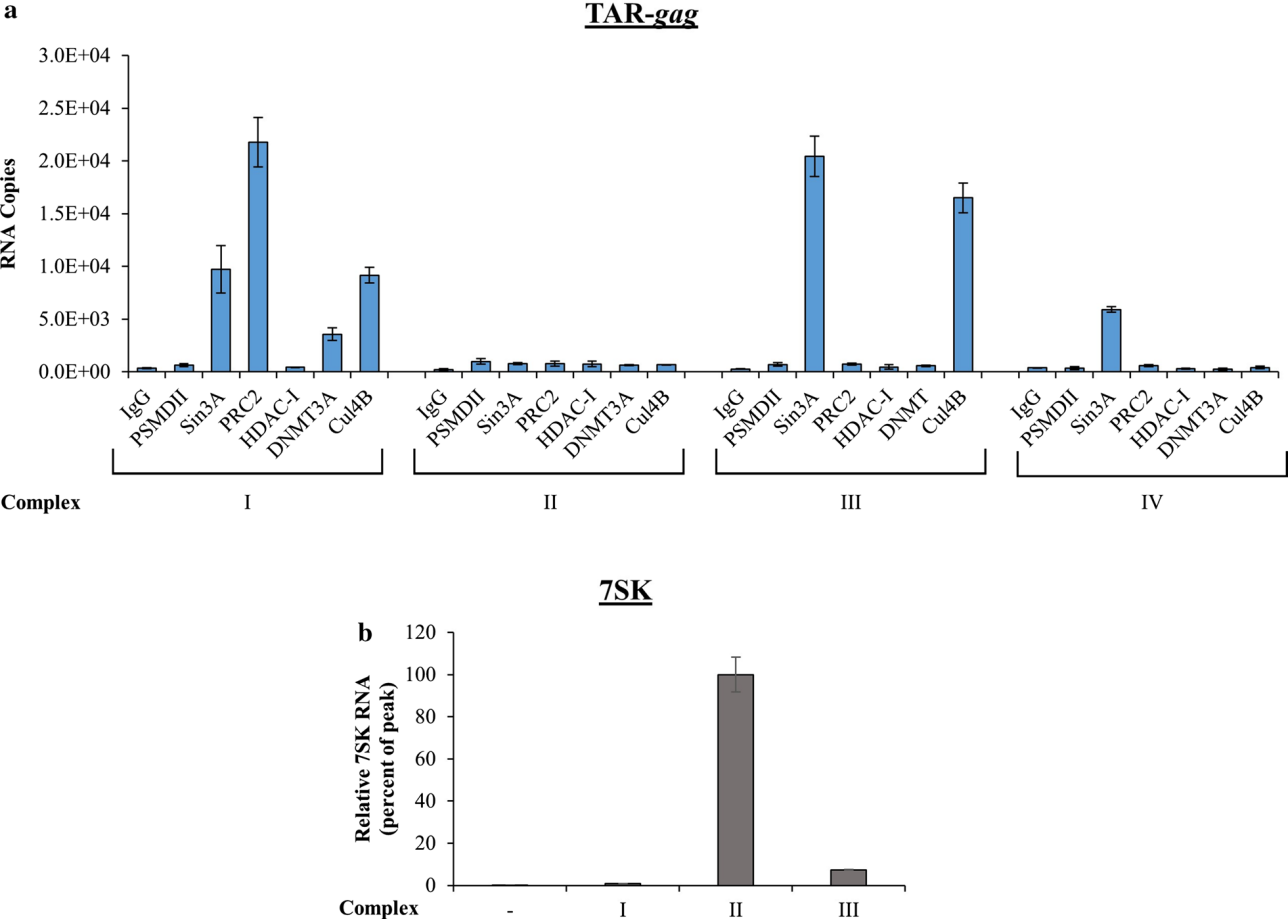
TAR-*gag* RNA association with various inhibitory complexes. **a** Early–mid log phase HIV-1 infected J1.1 cells were treated with F07#13 for 48 h (1 μM), pelleted, washed (×2) with PBS without Ca^2+^ and Mg^2+^, resuspended in lysis buffer, and 2500 µg of protein were equilibrated in degassed FPLC running buffer. A Superose 6 10/300 size-exclusion chromatography column was used to run lysed samples. Fractions were then pre-cleared with IgG for 2 h at 4 °C and then divided into 4 sub-fractions for IP using six antibodies against PSMD11, Sin3A, PRC2, HDAC-1, DNMT3A, and Cul4B (5 μg/reaction). Protein A/G was added the next day and the IPed complexes were washed. RNA was isolated for RT-qPCR using TAR-*gag* primers. An IP with IgG antibody was used as a control. Fractions from Complexes I, II, III, and IV constitute complex sizes from ~ 2.2 MDa to ~ 300 kDa. Error bars represent ± SD of three technical replicates. **b** Fractions from Complexes I, II, and III (500 µl) were nanotrapped with NT084 and assayed for RT-qPCR for presence of 7SK RNA. Fraction 10 was used as a control in lane 1 of this panel

We have recently shown that the HIV-1 promoter makes 4 distinct RNA molecules, all of which are non-coding [1, 7]. We used an RNA sequence analysis to define the 3′ ends of these 4 transcripts [1, 7], which are consistent with the presence of paused Pol II on the HIV-1 genome as shown by the Karn lab [36]. Our previous data has shown that these RNAs can exist intracellularly and extracellularly; however, the nature of the associated protein complexes are not known for the extracellular environment (exosomes from infected cells contain these RNA molecules) [1, 7]. As these RNA sequences all contain TAR RNA, they all have the potential to bind and sequester Tat protein. Here, we IPed from Flag-Tat_101_ transfected cells using previously established antibodies against proteins that complex with RNA and are responsible for TGS [1]. Following pre-clearing with IgG, we then used antibodies against PSMD11 (ubiquitin protein complex), Sin3A (responsible for binding to HDAC-1 and part of the suppressive SWI/SNF complex), PRCs (known RNA binding complexes containing EZH2), and Cul4B (ubiquitin complex marker) for our IPs. We then washed the complexes with TNE300 followed by TNE50 and isolated RNA for subsequent RT-qPCR RNA analysis. It is important to note that there were no cross-linking reagents used in these IPs. Results in Fig. 3a show that in F07#13 treated cells, TAR-*gag* was bound to PRC2, Sin3A, Cul4B, and low levels of DNMT3A in the large complex (Complex I). However, TAR-*gag* was mostly bound to Sin3A and Cul4B in the medium size complexes (Complex III). We also observed low levels of Sin3A binding to TAR-*gag* from Complex IV fractions. Interestingly, we have previously observed increased association between TAR-*gag*, HDAC-1, Sin3A, and PIWIL4 in F07#13 treated HIV-1 positive cells [1]. Nevertheless, these previous observations used whole cell extracts and not chromatographic separations as observed in Fig. 3a. Control 7SK RNA expression was also tested in these fractions, as we have previously shown that 7SK elutes mostly with complex II [53]. Results in Fig. 3b show the presence of 7SK in complex II, which is distinctly different from where TAR-*gag* elutes. Collectively, these results indicate that HIV-1 lncRNAs have the potential to bind to proteins that can regulate HIV-1 gene expression through an RNA–protein complex and potentially act as “RNA machines”.

### Presence of viral RNA protein complexes in PBMCs

**Fig. 4:**
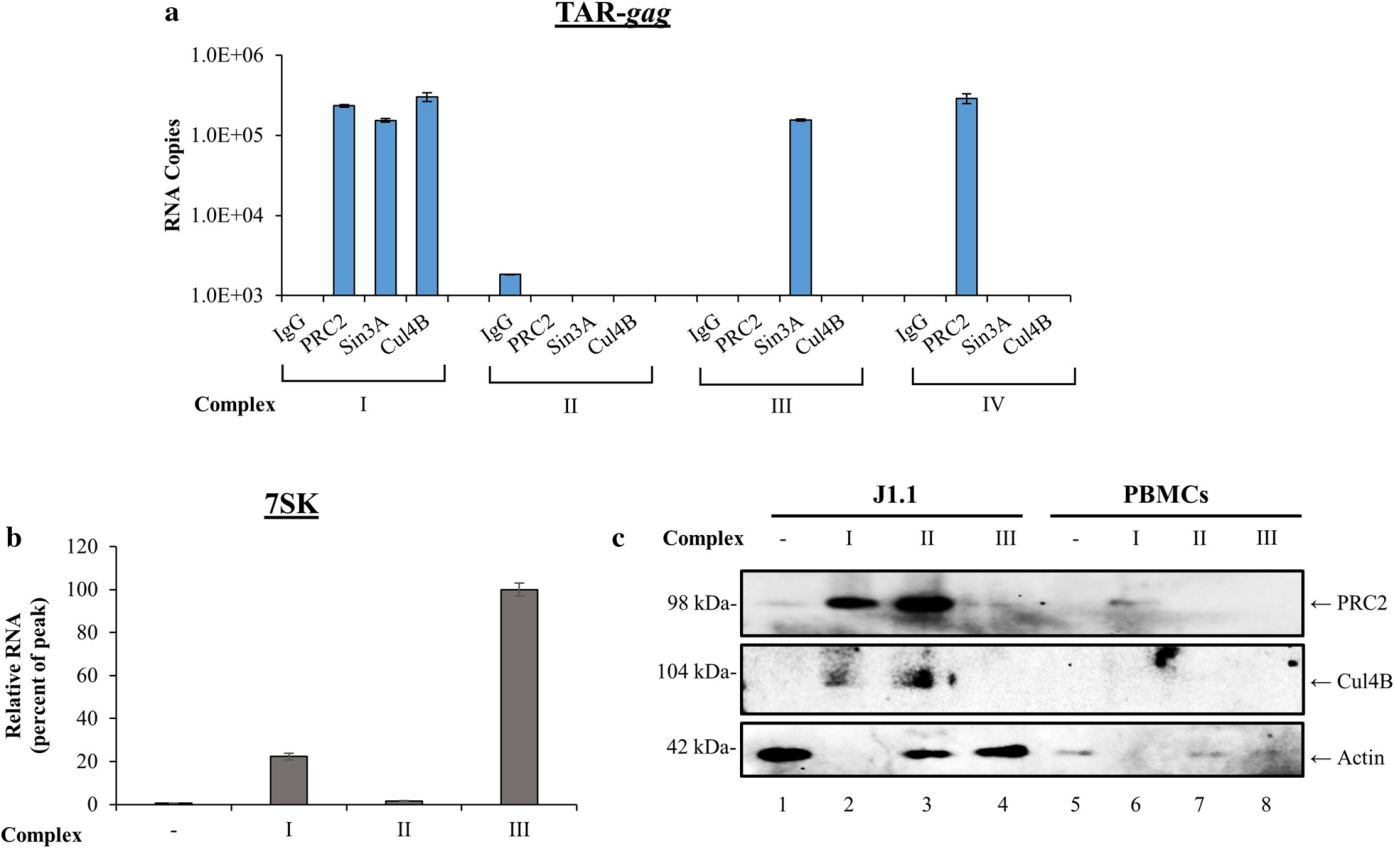
Presence of HIV-1 RNA associated complexes in multiple HIV-1 infected whole cell extracts. **a** Fresh primary PBMCs (10^7^ cells) were cultured with PHA/IL-2 for 7 days and infected with HIV-1 89.6 strain (MOI:1) [7]. Three days later they were treated with F07#13 (once every other day at 0.1 µM) for a total of 20 days. Cells were collected and lysates were loaded onto a sizing column under high salt. Column fractions were then IPed with antibodies against PRC2, Sin3A, Cul4B, and IgG. Following IP, RNA was isolated and samples were processed for RT-qPCR using primers against TAR-*gag*. Non-specific IgG background IPs were used as a control. Fractions from Complexes I, II, and III (500 µl) of infected PBMCs were nanotrapped with NT084 and assayed for RT-qPCR for presence of 7SK RNA (**b**) or half of the samples were run on an SDS/PAGE and Western blotted for presence of PRC2, Cul4B, actin, and Sin3A (data not shown) (**c**). Fraction 10 was used as a control in lane 1 of panels b and c. Error bars represent ± SD of three technical replicates

Here, we asked whether RNA from primary T-cells infected with dual tropic virus could still bind to some of the factors complexed with TAR-*gag*. We used a previously published procedure where fresh primary PBMCs (1 × 10^7^ cells) were cultured with PHA/IL-2 for 7 days and then infected with HIV-1 89.6 strain (MOI:1) [7]. Three days later they were treated with F07#13 (once every other day at 0.1 µM) for a total of 20 days. Cells were collected and lysates were loaded onto a sizing column under high salt. We then isolated specific fractions and IPed (250 µL aliquots) with either IgG, PRC2, Sin3A, or Cul4B (5 µg of each). Not enough material was obtained for IP against HDAC-1 or DNMT3A proteins. Following overnight IPs, complexes were collected using Protein A/G beads. RNA was isolated and RT-qPCR was performed for the presence of TAR-*gag*. Results in Fig. 4a show that there were specific complexes made in the large Complex I fraction where we observed PRC2, Sin3A, and Cul4B binding to TAR-*gag*. Complex II, similar to J1.1 fractions, did not contain appreciable amounts of RNA associated complexes; however, we observed the presence of Sin3A but no Cul4B in Complex III. Unlike the J1.1 extracts, we observed PRC2 binding in Complex IV in addition to Complex I. It is important to note that we do not know if these protein complexes are all on one RNA structure or if there are multiple forms of heterogeneous populations of RNA–protein complexes (i.e. Complex I). That would require further purification using mono-S and mono-Q columns or other more robust matrices, such as hydroxyapatite. Interestingly, the three proteins (PRC2, Sin3A, and Cul4B) complexed to TAR-*gag* RNA were all present in the higher molecular weight Complex I. We next performed RT-qPCR for the presence of 7SK RNA expression, and unlike J1.1, we observed some of the 7SK RNA present in Complex I, but mostly in Complex III (Fig. 4b). We currently do not understand the reasoning for this shift of the 7SK RNA into smaller complexes in primary cells infected with 89.6; however, we have observed presence of potentially two different RNAs in Complex I/III versus II, since the melting curve for the RNA in Complex II was slightly different than the other two complexes (83 °C vs. 85 °C; data not shown). Finally, we performed a similar pull-down experiment using NT084 from these fractions and Western blotted for presence of PRC2, Sin3A, and Cul4B. Data in Fig. 4c show that PRC2 (EZH2 subunit) was present in Complexes I and II in J1.1, as well as low levels of Cul4B in the same fractions. Results from PBMC Western blots were mostly unclear due to low protein recovery; however, we were able to observe a faint band for PRC2 in Complex I. Actin was used as a control for both cell types. Collectively, these data imply that TAR-*gag* maybe complexed with cellular proteins that normally regulate gene expression.

### Effect of F07#13 on HIV-1 LTR genome editing

We have recently shown that, contrary to widely-accepted latency models, the HIV-1 LTR is not a silent promoter, and Pol II is capable of transcribing through the LTR R/U5 region as well as the early stages of the *gag* gene between nucleosomes 2 and 3 in the presence of external signals such as exosomes [7]. This data was especially significant as it points toward an RNA polymerase that may alter the HIV-1 LTR DNA (by negative supercoiling, nucleosome remodeling, presence of various paused complexes, etc.), which may affect target recognition by guide RNA (gRNA) and ultimately gene editing. Therefore, our reasoning for performing these next set of experiments was that if the HIV-1 DNA is constantly occupied for transcriptional read-through resulting in production of ncRNA, then it may be difficult for the gRNA to find its target DNA, especially in the LTR, and allow subsequent DNA editing. Here, we asked whether F07#13 could potentially aid in pausing of Pol II to allow for a better gRNA recognition and gene editing.

**Fig. 5:**
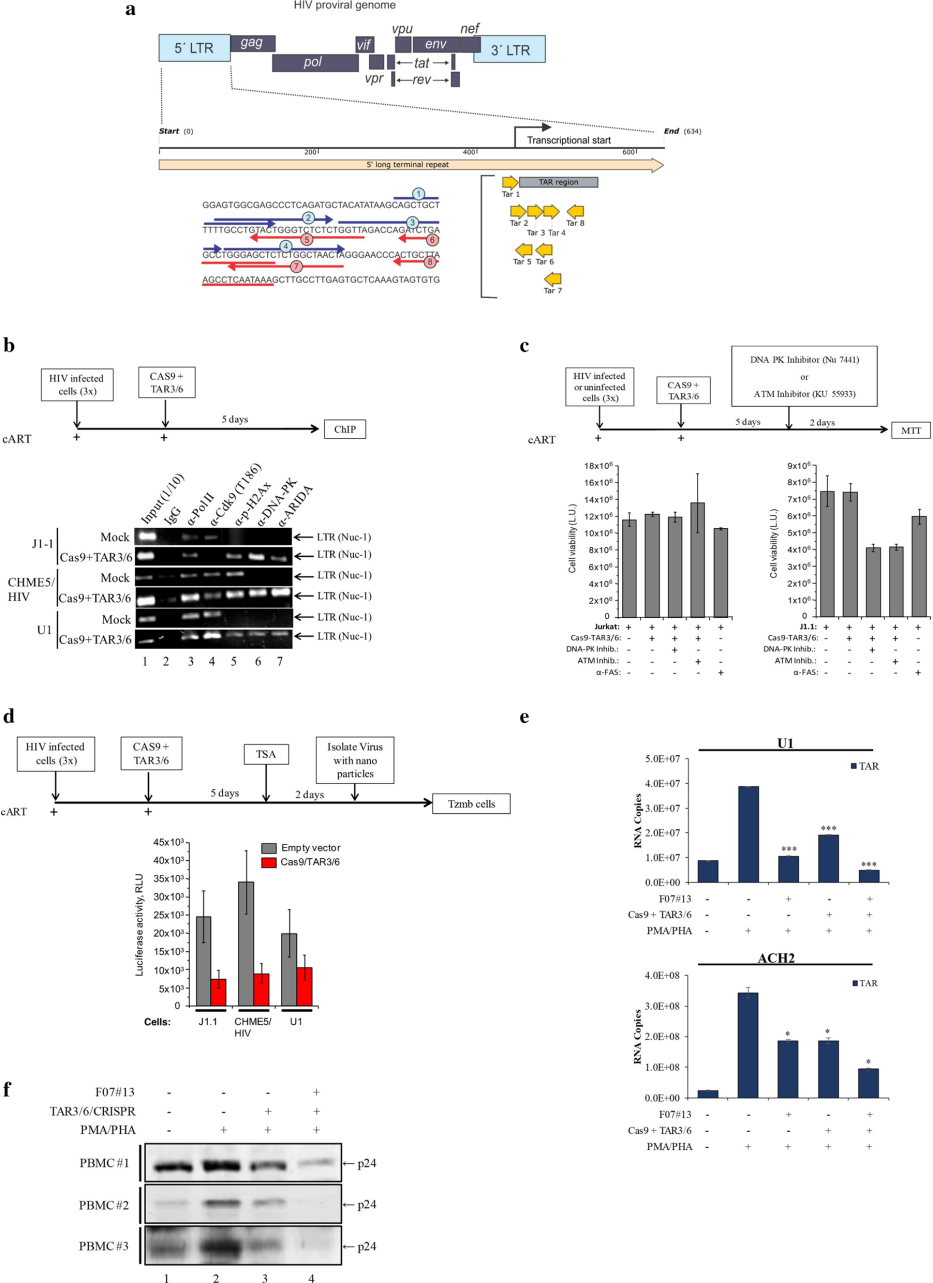
DNA-PK on the HIV-1 genome following Cas9+TAR3/6 transfection and alterations in cutting following F07#13 treatment. **a** Schematic of the HIV-1 proviral genome, which highlights the 5′ LTR of HIV-1. A series of gRNAs was designed to target the essential TAR loop required for Tat binding and proviral reactivation. **b** Three infected cell types (J1.1, CHME5/HIV, and U1) were grown in the presence of cART for 1 week prior to transfection. Cells were electroporated with three constructs at a 1:10 ratio (0.1 µg/1 µg of Cas9+TAR3/6) and kept in culture for 5 days. Approximately 1 × 10^7^ cells were used for ChIP assay using antibodies (10 µg) against Pol II large subunit, Cdk9 (T186), p-H2AX, DNA-PK, and ARIDA. Following DNA purification, samples were PCR amplified using LTR primers and run on a 2% Agarose gel. **c** Similar to panel b except cells were treated with two inhibitors after 5 days. Both inhibitors, DNA-PK inhibitor (Nu 7441, 0.2 µM) and ATM inhibitor (KU 55933, 1 µM), were used for a 2 day treatment of either uninfected (Jurkat) or infected (J1.1) cells prior to CellTiter-Glo. Positive control Fas antibody was used for apoptosis on both cell types. **d** Similar experimental design to panel b, except J1.1, CHEM5/HIV, and U1 cells were treated with 100 nM TSA after 5 days of transfection. Viruses were isolated from the supernatants with NT086 particles and added to TZM-bl-Luc cells. **e** A similar experiment as outlined in panel d; however, U1 and ACH2 cells were treated 1 day prior to PHA/PMA treatment with either F07#13 (Day 4), Cas9+TAR3/6, or both together and analyzed by RT-qPCR for the presence of TAR RNA. *p value ≤ 0.05; ***p value ≤ 0.001. **f** Latent PBMCs (3 independent donors) were created as described previously [7]. After cART/IL-7 addition, samples were divided into 4 sections; two were electroporated (210 V) with TAR3/6 DNA ± F07#13 and kept in culture for 4 days. They were then treated with PMA/PHA for 2 days prior to p24 Western blot

To perform these experiments, we first had to synthesize a series of gRNAs that would target the R region of the LTR. The vector system used caused double-stranded breaks guided by a 20-nt gRNA sequence within an associated CRISPR-RNA transcript [25]. To determine whether viral LTR could be targeted in infected latent cell lines, we treated three infected cell types with Cas9 and TAR gRNAs (gRNAs 1-8). Out of these 8 RNAs, we observed two gRNAs, gRNA 3 and 6, that showed partial editing (data not shown). Therefore, we focused on these two gRNAs for our subsequent set of experiments. The sequences and the directions of the gRNAs are shown in Fig. 5a.

We first asked whether TAR gRNA 3/6 (TAR3/6) vectors could show presence of DNA damage response proteins, such as DNA-PK, on the HIV-1 promoter. Here, we transfected J1.1, CHME-5/HIV [74], and U1 cells with Cas9 and TAR3/6 (1:10 ratio). All cells were treated with cART (10 µM) for 1 week prior to transfections to eliminate any residual virus. Cells were kept in culture for 5 days and subsequently cross-linked for chromatin immunoprecipitations (ChIPs) using various antibodies including α-Pol II, α-Cdk9 (T186), α-p-H2AX, α-DNA-PK, and α-ARIDA (Baf 250). Results in Fig. 5b show that mock treated cells contained Pol II and low levels of Cdk9 (T186) on the promoter. However, cells treated with triple plasmid (Cas9+TAR3/6) showed presence of p-H2AX, but more importantly DNA-PK and ARIDA, on the LTR. The presence of DNA-PK on the LTR is an indication of potential recruitment of DNA repair machinery, and the presence of ARIDA (a subunit of suppressive SWI/SNF complex) is an indication of suppressive chromatin involved in transcriptional silencing and potentially DNA repair.

We next asked whether inhibition of either DNA-PK or Ataxia telangiectasia mutated (ATM) kinase in Cas9 treated cells could result in apoptosis. Our rationale for these experiments was that if the HIV-1 genome is not properly repaired, then the cells might be pushed toward apoptosis. For this, we used inhibitors of ATM and DNA-PK that are being developed as potential therapeutics for the treatment of cancer [21]. Low concentrations of inhibitors for DNA-PK (Nu 7441) or ATM (KU 55933) were used in both infected and uninfected cells [29]. Results in Fig. 5c show that when infected cells are treated with either inhibitor, there is an increased level of apoptosis in infected T-cells but not in uninfected cells. Collectively, these results imply that the targeted Cas9 vectors may use either DNA-PK or ATM for repair and their inhibition pushes cells toward apoptosis.

We next performed a similar experiment as in Fig. 5b, but we added Trichostatin A (TSA) after 5 days to activate the latent viruses. The rationale for these experiments was that if the HIV-1 LTR genome was indeed mutated with Cas9+TAR3/6 constructs, then the resulting viral particles from these cells would be either non-infectious or contain particles with reduced infectivity. To assay for the released viruses, we utilized nanoparticles to trap and concentrate HIV-1 particles (NT086) and added the virus/nanoparticles onto the reporter TZM-bl-Luc cells [37]. Results in Fig. 5d indicate that viruses generated from latent cell lines released following Cas9+TAR3/6 treatment contained low levels (2–5 fold) of virus. Positive control experiments using no Cas9+TAR3/6 contained high levels of virus following induction with TSA from all tested cell types. Finally, we performed a similar experiment as outlined in Fig. 5d but treated cells 1 day prior to PHA/PMA treatment with F07#13 (Day 4). Here, the rationale was that if Pol II is paused following F07#13 treatment, then there would be a higher chance of gRNA finding its target DNA for editing, resulting in less viral product formation (i.e. Gag p24). Results of such an experiment are shown in Fig. 5e, where PHA/PMA treatment showed an increase in TAR RNA levels in myeloid and T-cells (U1: 3.9 × 10^7^ copies; ACH2: 3.4 × 10^8^ copies); a drop of RNA when using F07#13 (U1: 1.0 × 10^7^ copies; ACH2: 1.9 × 10^8^ copies); a drop of RNA when using Cas9+TAR3/6 (U1: 1.9 × 10^7^ copies; ACH2: 1.9 × 10^8^ copies); and a larger drop in RNA when using both F07#13 and Cas9+TAR3/6 (U1: 4.8 × 10^6^ copies; ACH2: 9.4 × 10^7^ copies). Finally, to test whether editing could take place in primary cells, we used a previously published latent model [7]. Three independent PBMCs were seeded at 10^7^ cells/mL and treated with PHA/IL-2 for 7 days. They were then infected with HIV-1 89.6 (MOI 10) for 3 days and treated with cART and IL-7 for another 9 days. The samples were divided into 4, out of which 2 were electroporated (210 V) with TAR3/6 vectors (20 µg) ± F07#13 (1 µM) and kept in culture for 4 days. They were then treated with PMA/PHA for 2 days prior to cell harvest and lysis. Cell lysates were then ran on a 4–20% gel for Western blot using anti-p24 antibody. Data in Fig. 5f show that all three PBMCs contained background levels of gene expression under these conditions (lane 1); however, upon addition of PMA/PHA, a robust gene expression was observed (lane 2). Samples that received TAR3/6 vectors were not as induced as the control (compare lane 3 to 2), and F07#13 treated cells showed minimal induction of gene expression and p24 levels (lane 4). Collectively, these data indicate that F07#13 may potentially slow down transcription, which would allow for better gene editing in these cells.

## Discussion

Today, HIV-1/AIDS patients primarily receive cART. This therapy works by targeting several steps of the viral life cycle including viral entry, reverse transcription, integration, and viral maturation. However, cART does not cure HIV-1 as it is unable to target latent viral reservoirs [60, 65, 77]. Additionally, there is currently no FDA-approved transcription inhibitor for the treatment of HIV-1. We have recently shown that this lack of a transcription inhibitor allows for the generation of TAR and TAR-*gag* transcripts [7]. This data is suggestive of paused polymerase sites located at nucleosome 1 and between nucleosome 2 and nucleosome 3, respectively. We have generated the 4 RNA structures based on 4 sequences of lengths + 1 to + 96 for Sequence I (TAR), + 1 to + 184 for Sequence II (TAR), + 1 to + 408 for Sequence III (TAR-*gag*), and + 1 to + 615 for Sequence IV (TAR-*gag*), and show the potential binding site for PRC2 in Sequences III and IV (Additional file 1: Fig. S2–S6). Importantly, when using F07#13, TAR-*gag* is significantly increased, but TAR levels were not [1]. We have previously shown that lower FPLC fractions (#15–30) presented the most noticeable increase in TAR-*gag*, suggesting TGS via blockage of elongation and increased protein recruitment by TAR-*gag* [1].

In the current manuscript, we asked whether F07#13 had secondary effects on latency. The primary mechanism of F07#13 was to disrupt Tat interaction with Cdk9/T1 complex and thereby stop or slow down Tat activated transcription [69]. However, we have consistently observed degradation of Tat in F07#13 treated cells, which was an unexpected finding. We suspected that Tat could be degraded through the ubiquitination and proteasome pathway. Here we have shown a dose-dependent decrease of Tat protein levels in cells treated with F07#13 (Fig. 1). Furthermore, when fractionating Tat associated complexes from F07#13 treated cells, we observed specific poly-ubiquitination of Tat from the large complex (Complex I) (Fig. 2), indicating that there was a selective processing of Tat in this complex and not the other Tat associated complexes.

**Fig. 6:**
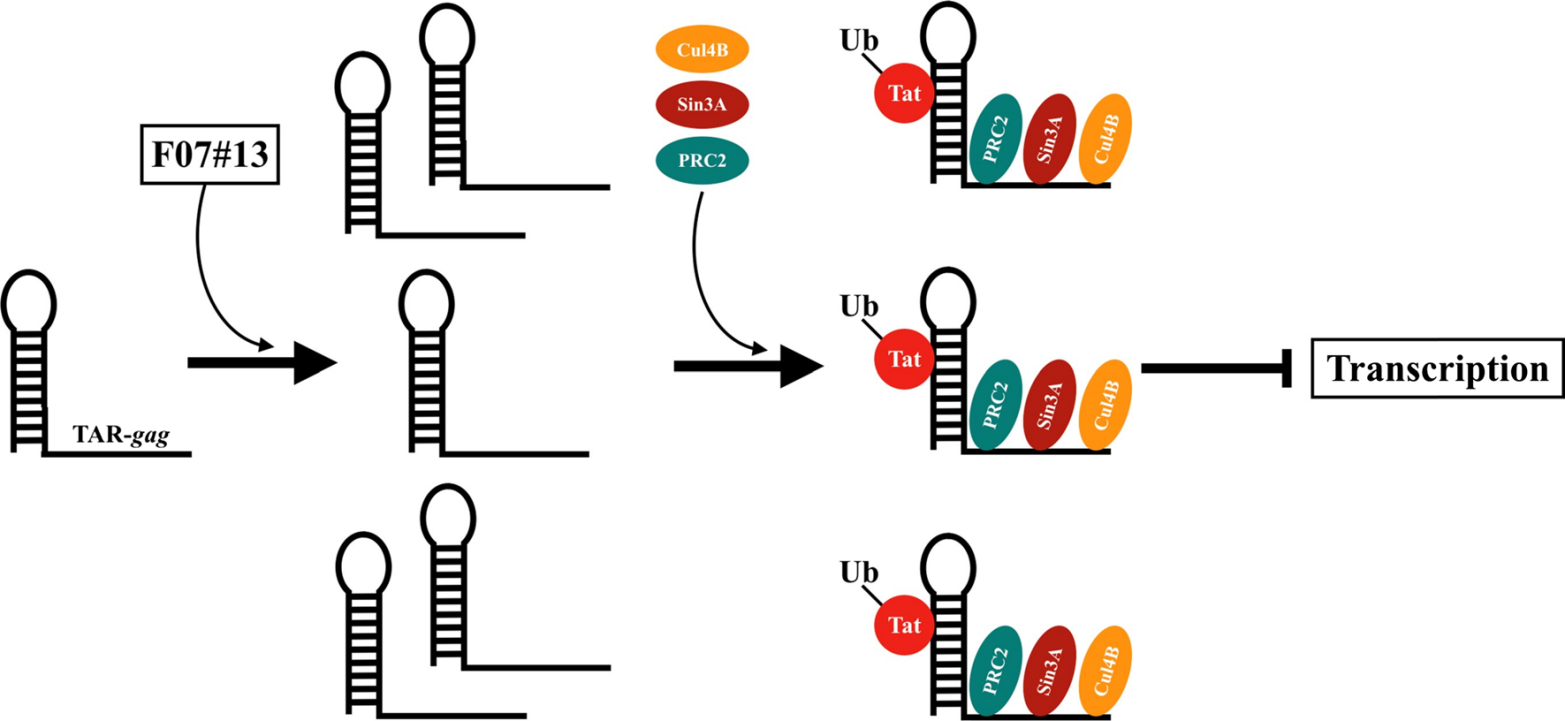
A proposed model of the effect of F07#13 on binding to TAR-*gag*. The model is based on the notion that the ncRNAs (i.e. TAR-*gag*) are made from HIV-1 LTR and upon the introduction of F07#13, there is an increase in the copy number of TAR-*gag* due to non-processive Pol II transcription. The increased abundance of TAR-*gag* leads to the sequestration of ubiquitinated Tat, potentially through the TAR sequence. The presence of protein complexes with RNA can constitute newly synthesized “RNA machines”, which cause the repression of HIV-1 transcription through epigenetic modifications and potentially contribute to gene silencing and latency

When performing RNA/protein IP, we found TAR-*gag*, a novel long HIV-1 ncRNA, associated with multi-suppressive protein complexes including Sin3A, PRC2, and Cul4B (Figs. 3, 4). Previous studies have shown the presence of similar RNA/protein complexes from plants [55]. This is also not surprising as mammalian PRC2, which is part of the polycomb complex, is able to bind to RNAs including HOTAIR, Xist, MEG3, ANRIL, PCAT, SChLAP1 (member of SWI/SNF), and ANCR [17, 50, 75]. Therefore, we suspect that one of the primary by-products of F07#13 treatment in infected cells is generation of HIV-1 ncRNA that is able to bind Tat (through TAR) and protein complexes (i.e. PRC2, Sin3A, and Cul4B) that may perform a number of functions including epigenetic regulation of either HIV-1 nascent RNA or viral DNA, modification of substrates such as histones (i.e. nucleosomes-1, 2, 3), and/or degradation of substrates such as Tat. Therefore, we collectively consider this new RNA/protein complex as part of an “RNA machine” (Fig. 6) that is mostly generated in the presence of transcription inhibitors. Future experiments using further purification followed by RNA/protein mapping will better determine the specificity of this interaction and its enzymatic activities, especially related to PRC2/RNA binding and Cul4B activity.

Using CRISPR/Cas9 technology against TAR, we found that 2 gRNAs were effective in binding to their target sites and editing the HIV-1 genome in three cell lines infected with LAI strains. These cell lines were of T-cell and myeloid origin. Using chromatin IP (ChIP) assays, we found that levels of p-H2AX, DNA-PK, and ARIDA proteins increased following Cas9+TAR3/6 treatment, which is an indication of repair of the genome. Importantly, the effect of editing increased with F07#13, indicating that the rate of Pol II loading and transcription may control the activities and efficiency of the gRNA targeting its site on the HIV-1 DNA (Fig. 5). Along these lines, future questions that still need to be addressed include: How does editing differ in active versus inactive sites of integration; does the HIV-1 copy number change (i.e. increase) over time with increasing number of defective viruses versus wild type virus following cART, thereby affecting editing; are there editing differences between infected central, transitional, and effector memory T-cells; and finally, what are the editing differences between T-cells and myeloid (i.e., macrophages, microglia, astrocytes) infected cells. Future in-depth experiments are currently in progress to address the efficacy of F07#13 inhibition in a humanized mouse model infected with dual-tropic 89.6 HIV-1 under cART and whether F07#13 treatment can contribute to CRISPR/Cas9 editing in blood and tissues. We have previously published the use of F07#13 in NOG animals and observed a significant drop of TAR RNA in animals activated with low level irradiation in blood, brain, and lung [1]; however we currently are expanding the number of animals to address the effect of the drug on gene editing in these tissues. These experiments are also being followed with mathematical modeling of short versus long transcripts in various tissues in the animals following treatment. Collectively, our data indicate that F07#13 not only inhibits Tat binding to Cdk9/T1 complexes but also contributes to transcriptional pausing and increase of viral ncRNAs (i.e. TAR and TAR-*gag*), which can then sequester Tat and aid in Tat degradation. It remains to be seen whether these effects can also be observed in various clades that have varying promoter strength, mutant LTRs, and in vivo.

## Conclusions

We conclude that the Tat peptide mimetic, F07#13, induces TGS of HIV-1 by induction of viral ncRNA (i.e. TAR and TAR-*gag*) and subsequent complexing with transcriptional suppressive proteins (i.e. PCR2, Sin3A, and Cul4B), promoting a TGS “RNA machine”, and may elicit Tat degradation by promoting Tat ubiquitination, resulting in inhibition of Pol II elongation. Furthermore, we report F07#13 synergizes with Cas9+TAR3/6 to impair HIV-1 replication in multiple cell types. These results provide insight into future potential uses of agents (i.e. F07#13) promoting formation of an “RNA machine” with specificity against HIV-1 transcription in clinical settings.

## Methods

### Cell culture and reagents

Uninfected T-cell (CEM), chronically HIV-1 infected T-cell lines (J1.1, 8E5, and ACH2), HIV-1 infected promonocytic cell (U1), as well as the promonocytic cell line (OM-10.1), were cultured in RPMI-1640 medium supplemented with 10% heat-inactivated FBS, 2 mM l-glutamine, 100 U/mL penicillin, and 100 µg/mL streptomycin. The J1.1 cell line is a Jurkat E6.1 derivative chronically infected with HIV-1 (LAI strain), while the ACH2 cell line was isolated from HIV-1 (LAV strain) infected A3.01 cells. TZMB cells were previously described [39]. HIV-1 89.6 plasmid is a dual-tropic strain. Cells infected with HIV-1 89.6 were treated for 7 days with a protease inhibitor (Indinavir) and nucleoside reverse transcriptase inhibitor (Emtricitabine) at a final concentration of 10 µM per drug. Both cell lines and antiretrovirals were obtained from the AIDS Reagent Program (National Institutes of Health).

### Antibodies

Antibodies used for Western blot were α-PSMD11 (Sigma; S1574); α-Sin3A antibody (Abcam, ab3479); α-PCR2 (EZH2) antibody (Cell signaling, 5246P); α-HDAC-1 (Abcam, ab7028); α-DNA PK (Abcam, ab18192); α-ARIDA (Santa Cruz, sc-32761, 1:250); α-CUL4B-A412 (Abcam, ab157103, 1:5000); anti-Dnmt3a (Abcam, ab2850); α-Ubiquitin antibody (Abcam, ab7780); Flag M2 antibody (Sigma, F1804), and α-Actin (Abcam, ab49900, 1:5000). Other antibodies used for this manuscript includedα-Pol II (Santa Cruz, sc-899, 1:250), α-p-Cdk9 (T-186) (Abcam, ab79178). α-p24 and α-Nef were obtained from NIH AIDS Reagent Program. Additionally, a cocktail of HIV-1 Tat Monoclonal (4D5.24), HIV-1 Tat Monoclonal (7D5.1), HIV-1 Tat Monoclonal (5A5.3), and HIV-1 Tat (NT3 2D1.1) was obtained from Dr. Jonathan Karn, also through the NIH AIDS Reagent Program. All other antibodies were used at a 1:1000 dilution.

### Transfection

The Cell-Porator™ [Life Technologies, Inc; Bethesda Research Labs (BRL)] was used to transfect cells per the manufacturer’s instructions. Briefly, Jurkat, J1.1, CHME5/HIV, and U1 cell lines were electroporated in RPMI 1640 media containing 10% FBS and 5% l-glutamine. The cell lines were transfected with DNA (20 µg) at the following parameters: a capacitance of 800 µF, low resistance, pulse voltage of 230 V for cell lines and 210 V for primary cells, and fast charge rate.

### Whole cell extract preparation and analysis by Western blot

Pellets from infected cells were collected and washed with phosphate-buffered saline (PBS). Subsequently, lysis buffer [50 mM Tris–HCl (pH 7.5), 120 mM NaCl, 5 mM EDTA, 0.5% Nonidet P-40, 50 mM NaF, 0.2 mM Na_3_VO_4_, 1 mM DTT, and 1 protease inhibitor cocktail tablet/50 mL (Roche Applied Science)] was used to resuspend pellets, which were then gently vortexed and incubated at 4 °C (or on ice) for 20 min with additional vortexing at every 5 min interval. Centrifugation (10,621×*g* for 10 min at 4 °C) was utilized to separate lysate from supernatant. Bradford protein assay (BioRad) was used to quantitate total protein concertation from collected lysates to be used for analysis by Western blot according to the manufacturer’s instructions. A mixture of Laemmli buffer with 20 μg of lysate was prepared (by gentle vortexing and heating at 95 °C for 3 min) and loaded onto a 4–20% Tris–glycine gel (Invitrogen) at a volume of approximately 10 µL for each sample. Western blot was run at 100 V until completed and followed by an overnight transfer at 50 mA onto PVDF Immobilon membranes (Millipore). A 2 h incubation at 4 °C with a mixture of 5% DIFCO™ Skim Milk (BD) in PBS with 0.1% Tween-20 (PBS-T) was used for blocking of non-specific antibody binding on PVDF membranes. Prior to adding primary antibody, a light rinse was performed with PBS-T to remove residual blocking solution. Corresponding primary antibodies were added and incubated with gentle rocking overnight at 4 °C. Secondary antibodies (HRP-conjugated) were added after three 5 min cycle wash steps with PBS-T and incubated with gentle rocking at 4 °C for 2 h. Western blots were developed by Clarity Western ECL Substrate (BioRad) and ChemiDoc Molecular Imager Touch system (BioRad) was used to visualize and capture images. ImageJ software was used to obtain raw densitometry counts. Counts were normalized to background.

### Isolation of RNA, generation of cDNA, and real-time quantitative PCR (RT-qPCR)

Whole cell lysates were used as sources of total RNA and later separated by Trizol-chloroform (MRC) per the manufacturer’s instructions. Subsequently, specific reverse primers and GoScript Reverse Transcription System (Promega) were used to yield the corresponding cDNA from RNA isolates. Additionally, cDNA was also generated from purified total RNA obtained by NT086 nanotrap (Ceres Nanosciences Inc.) bound to virus. The following reserve primers were used: TAR Reverse: (5′-CAA CAG ACG GGC ACA CAC TAC-3′, Tm = 58 °C), Gag Reverse: (5′-GCT GGT AGG GCT ATA CAT TCT TAC-3′; Tm = 54 °C), and OligoDT. Next, real time quantitative polymerase chain reaction (RT-qPCR) analysis was performed with 2 μL of undiluted aliquots of cDNA using iQ supermix (BioRad) with primers specific for target TAR sequences: TAR-Reverse: (5′-CAA CAG ACG GGC ACA CAC TAC-3′, Tm = 58 °C) and TAR-Forward (5′-GGT CTC TCT GGT TAG ACC AGA TCT G-3′, Tm = 60 °C); TAR-Probe (5′-/56-FAM/AGC CTC AAT AAA GCT TGC CTT GAG TGC TTC/36-TAMSp/-3′; Tm = 63.2 °C); GAPDH-Reverse (5′-CAG AGT TAA AAG CAG CCC TGG T-3′, Tm = 57.5 °C); GAPDH-Forward (5′-GAA GGT GAA GGT CGG AGT CAA C-3′, Tm = 57.5 °C); GAPDH-Probe (5′-/56-FAM/TTT GGT CGT ATT GGG CGC CT/36-TAMSp/-3′, Tm = 59.8 °C). For RT-qPCR of 7SK RNA, total RNA was isolated from fraction pull-downs with NT084 and generated corresponding cDNA with GoScript Reverse Transcription System (Promega, Madison, WI) using 7SK-RT reverse primer (5′-AAAAGAAAGGCAGACTGCC-3′). Relative RNA abundance (percent of peak) was quantitated by RT-qPCR using SYBR Green (BioRad, Hercules, CA) with the following pair of primers: 7SK-F (5′-GACA TCTGTCACCCCATTGA-3′) and 7SK-R (5′-GCGCAGCTACTCGTATACCC-3′).

7SK RNA is shown as a percentage relative to the highest peak for each cell type. DNA from 8E5 cells (CEM T-cell line containing a single copy of HIV-1 LAV provirus per cell) was used as the quantitative standard after obtaining a concentration gradient by serial dilutions. The following PCR settings were used for TAR/TAR-*gag*: one cycle at 95 °C for 2 min, 41 cycles at 95 °C for 15 s, and 60 °C or 50 °C (for respective primer sets) for 40 s. PCR settings for 7SK RNA were the following: one cycle at 50 °C for 2 min, followed by 95 °C for 2 min, 41 cycles at 95 °C for 30 s, 65 °C for 30 s, and 72 °C for 30 s. The limit of detection (LoD) was assessed by quantitation of background TAR-*gag* RNA copies detected in negative control (DiH20) and was determined to be 0 copies, also an indication of the specificity of our primer sequence. However, the limit of quantitation (LoQ) was established by our lowest concentration standard at 8 × 10^1^ RNA copies of 8E5 cells. The cycle threshold (Ct) value relative to the standard curve and used for absolute quantitation of samples. The BioRad CFX96 Real Time System was used for RT-qPCR. All RT-qPCR experiments were run in technical triplicates.

### Chromatin immunoprecipitation (ChIP)

Cells were harvested and ChIP assay was performed with the Imprint Chromatin Immunoprecipitation Kit (Sigma) per the manufacturer’s instructions. Briefly, samples were crosslinked, quenched, sonicated, and mono-disomes were used for immunoprecipitation. Appropriate antibodies were added, and the samples rotated overnight at 4 °C. A 50% (v/v) protein A-Sepharose/protein G-Sepharose mix was added, and the samples were rotated for 2 h at 4 °C. The samples were washed two times with IP Wash Buffer (Sigma) before addition of Proteinase K (800 U/mL), then subsequently incubated for 15 min at 65 °C. Reversing solution (Sigma) was added and the samples were incubated at 65 °C for 90 min. DNA was purified using elution columns and qPCR was performed with appropriate primers.

### Cell viability

Cell viability was assessed by plating 5 × 10^4^ cells (grown in fresh RPMI media and supplemented as described above) into a 96-well cell culture plate for 7 days, followed by treatment with Cas9+TAR3/6. Following a 5-day incubation, cells were treated with either DNA-PK inhibitor (NU 7441, SelleckChem, S2638), ATM inhibitor (KU 55933, SelleckChem, S1092), FAS antibody (Santa Cruz, sc-715), or a combination thereof. After 2 days, cell viability was tested using CellTiter-Glo Luminescent Cell Viability Assay (Promega). Luminescence was measured using the GloMax Multi-Detection System (Promega). All cell viability assays were conducted in biological triplicate and background signal was normalized with fresh RPMI media.

### Size-exclusion chromatography

A transfection with Flag-Tat_101_ (a generous gift from Dr. Kuan-Teh Jeang at National Institutes of Health) was performed on HIV-1-infected (J1.1) and uninfected (Jurkat and CEM) cells at the early-mid log phase, and cells were then treated with F07#13 for 48 h. Pellets were then collected, washed with PBS, and lysed as described previously (“Effects of F07#13 on various Tat complexes” section). Protein concentrations for supernatants were quantified via Bradford protein assay (BioRad) and 2.5 mg of each sample was then equilibrated in 0.2 M Tris–HCl (pH 7.5), 0.5 M NaCl, and 5% glycerol. Samples underwent fast protein liquid chromatography (FPLC) (ÄKTA Purifier system; GE Healthcare Bio-Sciences) on a Superose 6 10/300 size-exclusion column (GE Healthcare Bio-Science). Separation between lower-molecular-weight complexes, eluting in higher fractions (far right-side of the column), and high-molecular-weight fractions, eluting in lower fractions (far left-side of the column), was improved by introducing a quarter-inch gap at the top of the Superose 6 column. Flow rate parameters for the FPLC were set at 0.3 mL/min and 0.5 mL fractions of the flow-through were collected at 4 °C with approximately 60 fractions per sample (1 mL injected). Nanotrap (NT084; Ceres Nanosciences Inc.) capture was used on every fifth fraction. Nanotrapped pellets were centrifuged at 4 °C for 10 min at 15,294×*g*, supernatants were removed, and the pellets were allowed to dry for a few minutes at room temperature. The pellets were resuspended in Laemmli buffer and analyzed by immunoblotting for Tat as needed [a cocktail of HIV-1 Tat Monoclonal (4D5.24), HIV-1 Tat Monoclonal (7D5.1), HIV-1 Tat Monoclonal (5A5.3), and HIV-1 Tat (NT3 2D1.1) from Dr. Jonathan Karn, obtained through the AIDS Research and Reference Reagent Program, Division of AIDS, NIAID, NIH], cyclin T1 (Santa Cruz Biotechnology Inc., H-245), Cdk9 (Santa Cruz Biotechnology Inc., C-20), and β-actin (Abcam, AB49900). Additionally, every fifth fraction from J1.1 cells that were electroporated with Flag-Tat_101_ was screened for the elution of Tat using Flag M2 antibody.

### Immunoprecipitation

Immunoprecipitation (IP) of RNA/protein complexes were performed by incubation of 0.5–1 mL of lysate fractions with 10 µg of primary antibody overnight. The next day, a 30% slurry of Protein A/G beads (Calbiochem) was added to the immunocomplexes and allowed to incubate for 2 h at 4 °C. Samples were then washed twice with PBS and prepared for downstream assays. For the preparation of IP samples for RNA isolation, samples were resuspended in 50 µL of PBS and subjected to RNA isolation for downstream RT-qPCR. In preparation for Western blot, resulting immunocomplexes were added to 10 µL of Laemmli Buffer and subjected to the Western blot protocol described above.

### Generation of Cas9+TAR3/TAR6 vectors

A generic sgRNA expression vector with a BsmBI cloning site was synthesized as gBLOCKs (IDT), which was amplified using a U6-F (5′-GAGGGCCTATTTCCCATG-3′) and a tracrRNA-R primer (5′-ATAGCTTCCACCGCGGTGGCACGCGTAAAAAAGCACC GACTCGGTGCCACTTTTTCAAGTTGATAACGGACTAGC-3′) with KAPA Taq Readymix (Roche Applied Science) and ligated into a pTZ57R/T vector using a InsTAclone PCR Cloning Kit (Thermo Scientific) according to the manufacturer instructions. To generate the TAR sgRNAs, the pTZ-U6-BsmBI-sgRNA backbone was prepared by digestion with BsmBI and the sgRNA target sequences were ligated into the vector using a standard complementary oligomer protocol (IDT). The pTZ-U6-BsmBI-sgRNA and pTZ-U6-sgRNAs TAR1-8 were confirmed using automated sequencing. The *sp*Cas9 expression vector was obtained from Addgene (px330, #42230).

### Prediction of RNA secondary structure

For prediction of the RNA secondary structures, the sequence of HIV-1 LTR and p17 region of *gag* DNA sequences were submitted to the Vienna RNA secondary structure server [28, 31]. The server predicts the minimum free energy *(mfe)* secondary structures for single RNA sequences/DNA sequences. The MFE structure of an RNA sequence is the secondary structure that contributes a minimum of free energy. This structure is predicted using a loop-based energy model and the dynamic programming algorithm introduced by Zuker et al. [78]. This server also calculates the full equilibrium partition function for secondary structure and the probabilities of various substructures by using partition function (*pf*) algorithm proposed by McCaskill [49]. All the secondary structure predictions were performed for a temperature of 37 °C, keeping all the other parameters to default [46].

### Statistical analysis

Standard deviations (SD) were calculated using Microsoft Excel for every quantitative experiment. Two-tailed student’s *t*-test were performed to obtain *p*-values to determine statistical significance. Values could be considered statistically significant (*p *< 0.05), of greater significance (*p *< 0.01), and of greatest significance (*p *< 0.001).

## Additional file

**Additional file 1.** Supplementary Data.

## Abbreviations

AIDS: acquired immunodeficiency syndrome
cART: combination antiretroviral therapy
Cas9: CRISPR associated protein 9
Cdk: cyclin dependent kinase
ChIP: chromatin immunoprecipitation
CMV: cytomegalovirus
CRISPR: clustered regularly interspaced short palindromic repeats
DMA: dimethylacetamide
FPLC: fast protein liquid chromatography
gRNA: guide RNA
HAND: HIV-1 associated neurocognitive disorder
HIV-1: human immunodeficiency virus 1
i.p.: intraperitoneal
IFN-γ: interferon-gamma
IP: immunoprecipitation
IR: ionizing radiation
lncRNAs: long non-coding RNA
LoD: limit of detection
LoQ: limit of quantitation
LTR: long terminal repeat
miRNA: micro RNA
ncRNA: non-coding RNA
nuc: Nucleosome
P-TEFb: positive transcription elongation factor b
PBS-T: PBS-Tween 20
PBS: phosphate-buffered saline
PD: pharmacodynamics
PEG: polyethylene glycol 400
PHA: phytohemagglutinin P
PK: pharmacokinetics
PMA: phorbol 12-myristate 13-acetate
Pol II: RNA polymerase II
RT-qPCR: real-time quantitative polymerase chain reaction
SEC: super elongation complex
snRNA: small nuclear RNA
TAR: transactivating response element
TCR: T cell receptor
TGS: transcriptional gene silencing
Ub: ubiquitinated
