# Supplementary material for: Effect of transcription inhibition and generation of suppressive viral non-coding RNAs
Source: Retrovirology. 2019 Apr 29;16:13. doi: 10.1186/s12977-019-0475-0 (PMC6489247; doi:10.1186/s12977-019-0475-0)
Supplement: Supplementary file 1 — Additional file 1. Supplementary Data. [file 12977_2019_475_MOESM1_ESM.pdf]

Effect of Transcription Inhibition and Generation of Viral Non-coding RNAs

Daniel O. Pinto[1], Tristan A. Scott[2], Catherine DeMarino[1], Michelle L. Pleet[1], Thy T. Vo[1], Mohammed Saifuddin[1], Dmytro Kovalskyy[3], James Erickson[1], Maria Cowen[1], Robert A. Barclay[1], Chen Zeng[4], Marc S. Weinberg[5,6], Kevin V. Morris[2], Fatah Kashanchi[1*]

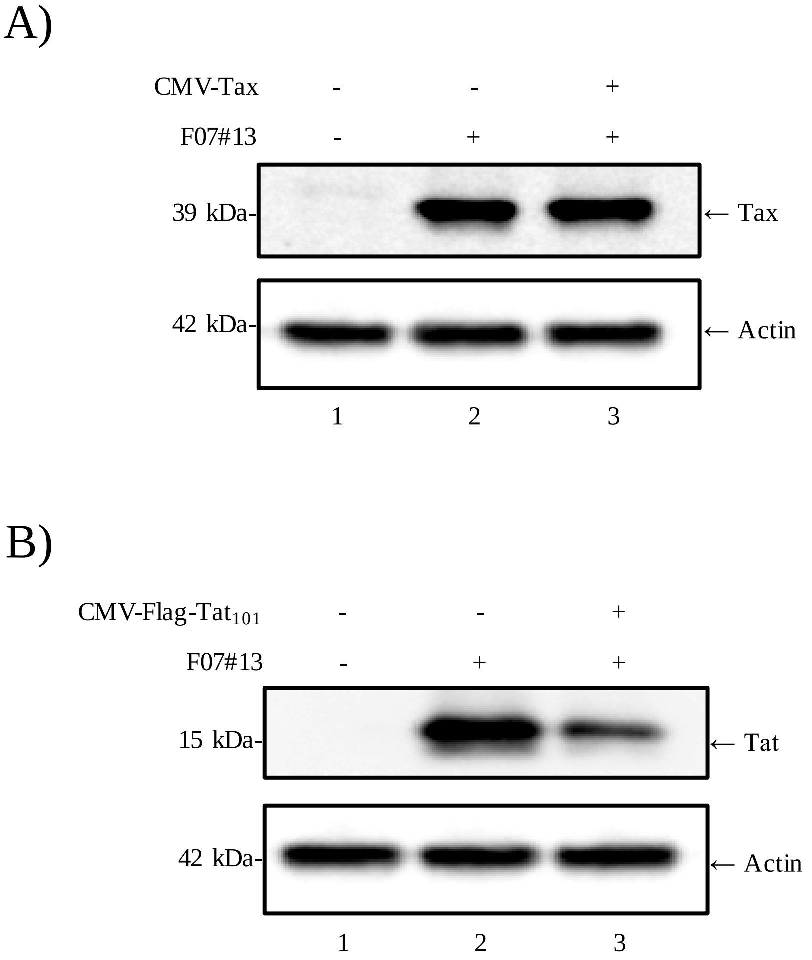

Figure S1A, B: Effect of F07#13 on Tax and Tat proteins. **A)** CMV-Tax (20 µg) was transfected into Jurkat cells and treated with F07#13 (1µM) for 48 hrs. Samples were collected, washed, and lysates were run on a 4-20% gel for western blots. **B**) CMV- Flag-Tat_{101} (Flag-Tat_{101}; 20 µg) was transfected into Jaurkat cells and treated with F07#13 (1µM). Samples were washed and processed for western blot with anti- Tax (Tab 169, 170, 172) for panel A and anti-Tat polyclonal rabbit antibody (NIH-ARP, Cat 705). Actin was used as control.

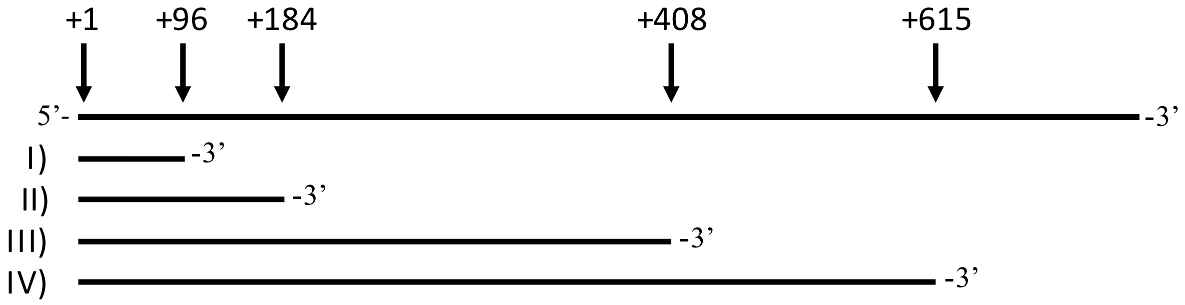

Figure S2: Lengths of TAR-*gag*. In our 2017 publication (Barclay, et al., 2017), we performed RNA sequencing to determine the length of a novel HIV-1 RNA, TAR-*gag*. Four clusters of RNAs were found, terminating at 96 nt, 184 nt, 408 nt, and 615 nt past the transcription start site (+1).

Sequence 1:
GGTCTCTCTGGTTAGACCAGATCTGAGCCTGGGAGCTCTCTGGCTAA
CTAGGGAACCCACTGCTTAAGCCTCAATAAAGCTTGCCTTGAGTGCTTC

The free energy of the thermodynamic ensemble is **-38.50** kcal/mol.

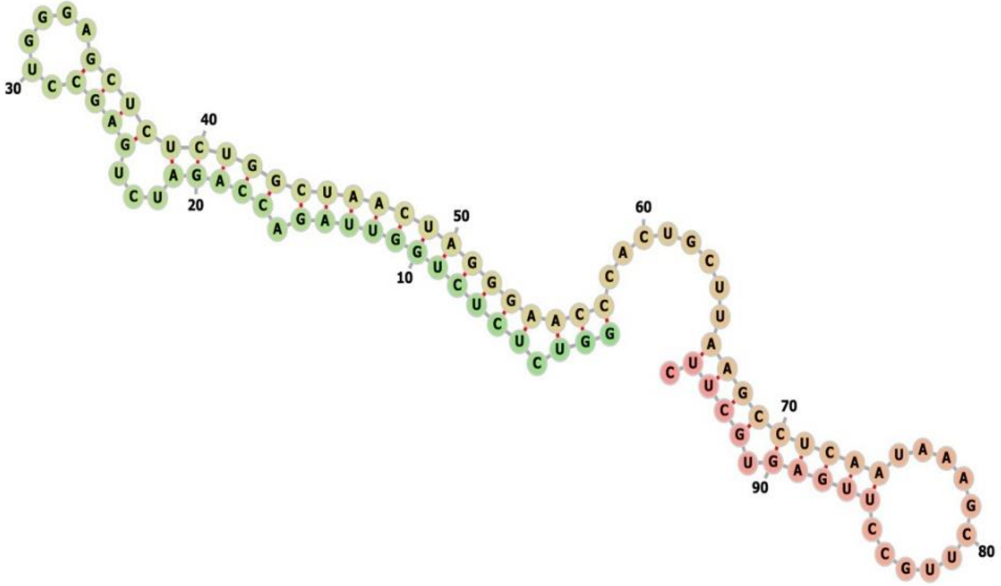

Fig. S3: TAR-*gag* Cluster 1. Secondary structure of the first TAR-*gag* construct, generated by Vienna RNA secondary structure server, which is 96 nt in length. The DNA sequence and the RNA product is shown above.

Sequence 2:
GGTCTCTCTGGTTAGACCAGATCTGAGCCTGGGAGCTCTCTGGCTAACTAGGGAACCCACTGCTTAAGCCTCAATAAAGCTT
GCCTTGAGTGCTTCAAGTAGTGTGTGCCCGTCTGTTGTGTGACTCTGGTAACTAGAGATCCCTCAGACCCTTTTAGTCAGTG
TGGAAAATCTCTAGCAGTGG

The free energy of the thermodynamic ensemble is **-69.30** kcal/mol.

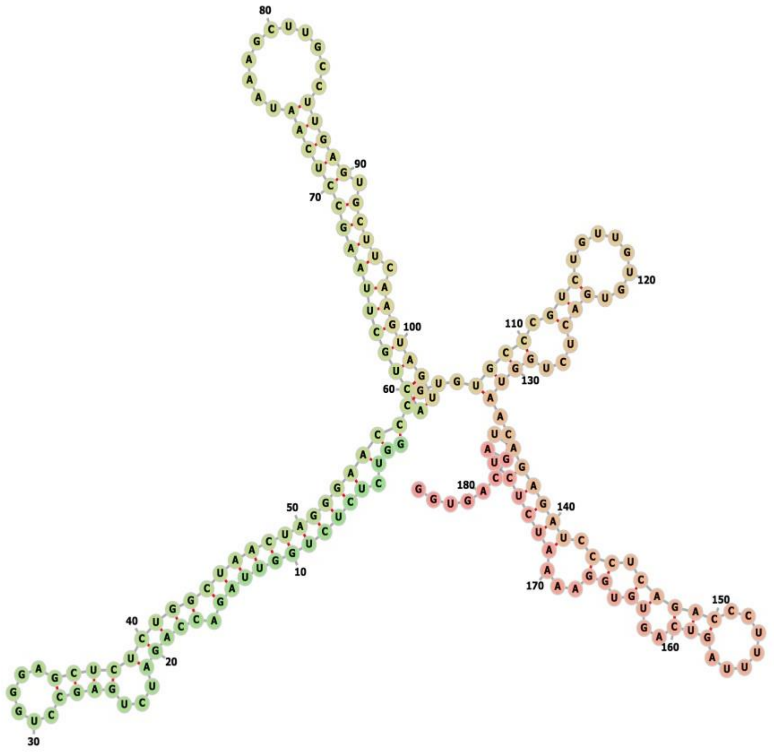

Fig. S4: TAR-*gag* Cluster 2. Secondary structure of the second TAR-*gag* construct, generated by Vienna RNA secondary structure server, which is 184 nt in length. The DNA sequence and the RNA product is shown above.

Sequence 3:
GGTCTCTCTGGTTAGACCAGATCTGAGCCTGGGAGCTCTCTGGCTAACTAGGGAACCCACTGCTTAAGCCTCAATAAAGCTTGCCTTGAGTGCTTCAAG
TAGTGTGTGCCCGTCTGTTGTGTGACTCTGGTAACTAGAGATCCCTCAGACCCTTTTAGTCAGTGTGGAAAATCTCTAGCAGTGGGCCCGAACAGGGAC
CTGAAAGCGAAAGGGAAACCAGAGGAGCTCTCTCGACGCAGGACTCGGCTTGCTGAAGCGCGCACGGCAAGAGGCGAGGGGCGGCGACTGGTGA
GTACGCCAAAAATTTTGACTAGCGGAGGCTAGAAGGAGAGAGATGGGTGCGAGAGCGTCAGTATTAAGCGGGGGAGAATTAGATCGATGGGAAAAA
ATTCGGTTAAGGCCAGGGGG

The free energy of the thermodynamic ensemble is **-153.63** kcal/mol.

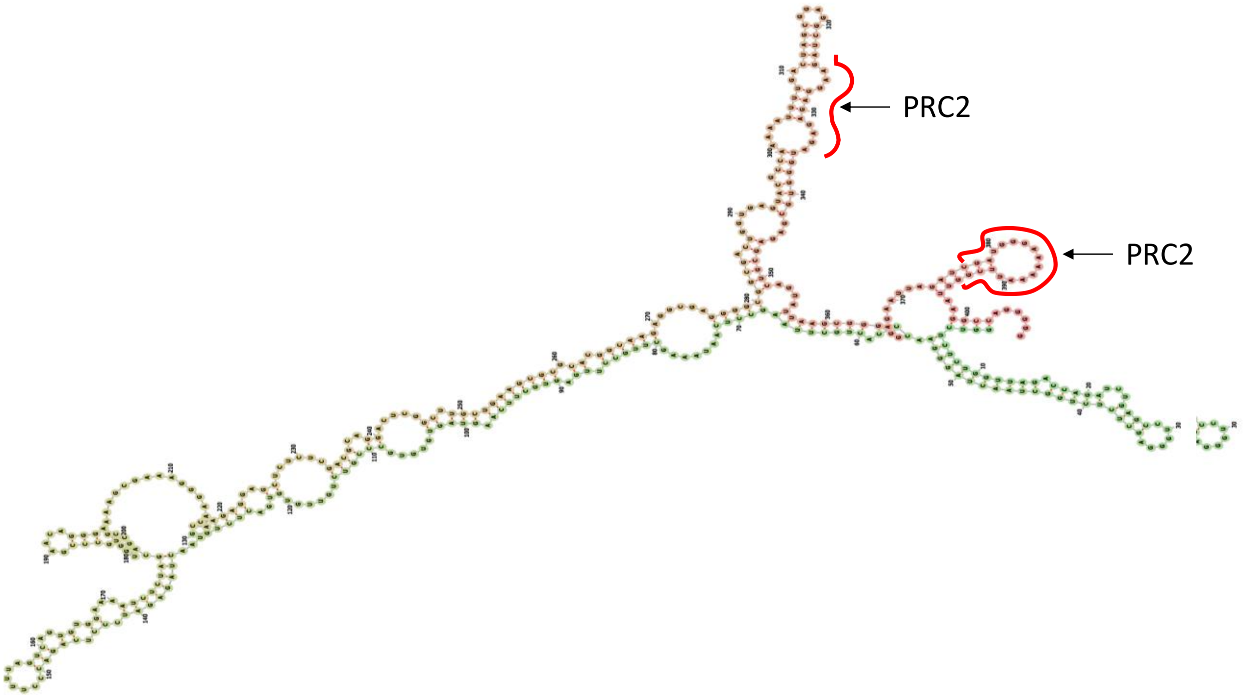

Fig. S5A: TAR-*gag* Cluster 3. Secondary structure of the third TAR-*gag* construct, generated by Vienna RNA secondary structure server, which is 408 nt in length. This RNA includes two potential binding motifs for PRC2, which is identified by the red line. The DNA sequence and the RNA product is shown above.

Sequence 4:
GGTCTCTCTGGTTAGACCAGATCTGAGCCTGGGAGCTCTCTGGCTAACTAGGGAACCCACTGCTTAAGCCTCAATAAAGCTTGCCTTGAGTGCTTCAAGTAGTGTGTGCCC
GTCTGTTGTGTGACTCTGGTAACTAGAGATCCCTCAGACCCTTTTAGTCAGTGTGGAAAATCTCTAGCAGTGGCGCCCGAACAGGGACCTGAAAGCGAAAGGGAAACCA
GAGGAGCTCTCTCGACGCAGGACTCGGCTTGCTGAGCGCGCACGGCAAGAGGCGAGGGGCGGCGACTGGTGAGTACGCCAAAAATTTTGACTAGCGGAGGCTAGAAG
GAGAGAGATGGGTGCGAGAGCGTCAGTATTAAGCGGGGGAGAATTAGATCGATGGGAAAAAATTCGGTTAAGGCCAGGGGGAAAGAAAAAATATAAATTAAAACATA
TAGTATGGGCAAGCAGGGAGCTAGAACGATTCGCAGTTAATCCTGGCCTGTTAGAAACATCAGAAGGCTGTAGACAAATACTGGGACAGCTACAACCATCCCTTCAGAC
AGGATCAGAAGAACTTAGATCATTATATAATACAGTAGCAACCCTCTATTGTGTGCATCAAAGGATAGAGAT

The free energy of the thermodynamic ensemble is **-195.78** kcal/mol
.

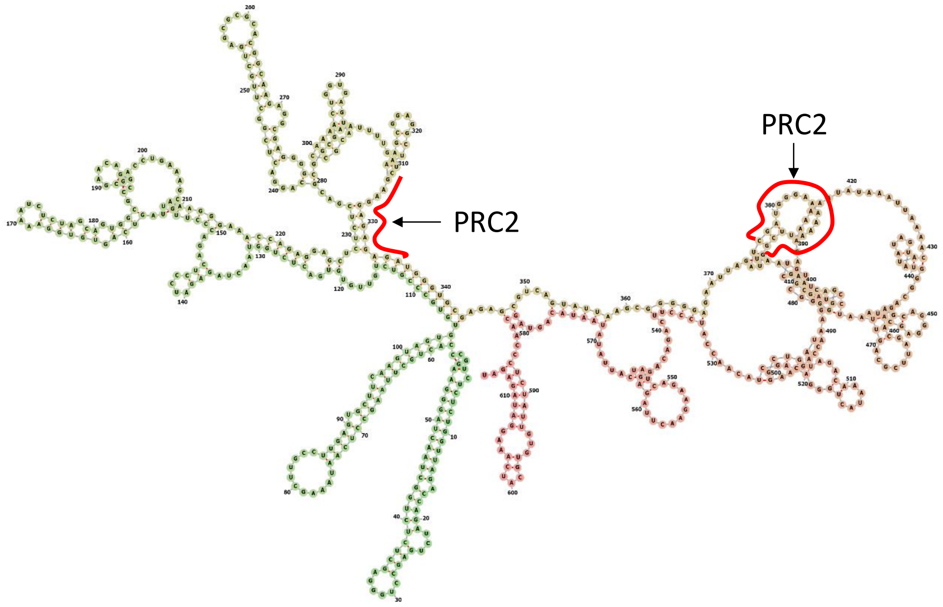

Fig. S6: TAR-*gag* Cluster 4. Secondary structure of the fourth TAR-*gag* construct, generated by Vienna RNA secondary structure server, which is 615 nt in length. This RNA includes two potential binding motifs for PRC2, which is identified by the red line. The DNA sequence and the RNA product is shown above.
